# Semi-supervised consensus clustering for gene expression data analysis

**DOI:** 10.1186/1756-0381-7-7

**Published:** 2014-05-08

**Authors:** Yunli Wang, Youlian Pan

**Affiliations:** 1 National Research Council Canada, 46 Dineen Dr., Fredericton, Canada; 2 National Research Council Canada, 1200 Montreal Rd., Ottawa, Canada

**Keywords:** Semi-supervised clustering, Consensus clustering, Semi-supervised consensus clustering, Gene expression

## Abstract

**Background:**

Simple clustering methods such as hierarchical clustering and *k*-means are widely used for gene expression data analysis; but they are unable to deal with noise and high dimensionality associated with the microarray gene expression data. Consensus clustering appears to improve the robustness and quality of clustering results. Incorporating prior knowledge in clustering process (semi-supervised clustering) has been shown to improve the consistency between the data partitioning and domain knowledge.

**Methods:**

We proposed semi-supervised consensus clustering (SSCC) to integrate the consensus clustering with semi-supervised clustering for analyzing gene expression data. We investigated the roles of consensus clustering and prior knowledge in improving the quality of clustering. SSCC was compared with one semi-supervised clustering algorithm, one consensus clustering algorithm, and *k*-means. Experiments on eight gene expression datasets were performed using *h*-fold cross-validation.

**Results:**

Using prior knowledge improved the clustering quality by reducing the impact of noise and high dimensionality in microarray data. Integration of consensus clustering with semi-supervised clustering improved performance as compared to using consensus clustering or semi-supervised clustering separately. Our SSCC method outperformed the others tested in this paper.

## Background

Simple clustering methods such as agglomerative hierarchical clustering and *k*-means have been widely used on gene expression data analysis. However, individual clustering algorithms have their limitations in dealing with different datasets. For example, *k*-means is unable to capture clusters with complex structures, and selection of *k* value is somewhat challenge without subjectivity. Therefore, many studies used consensus clustering (also called cluster ensemble) to improve the robustness and quality of clustering results [1-4].

Consensus clustering solves a clustering problem in two steps. The first step, known as base clustering, takes a dataset as input and outputs an ensemble of clustering solutions. The second step takes the cluster ensemble as input and combines the solutions through a consensus function, and then produces final partitioning as the final output, known as final clustering. The consensus clustering algorithms differ in chosen algorithms for basic clustering, consensus function and final clustering. Monti et al. used hierarchical clustering(HC) or self-organizing map (SOM) as the base clustering to generate consensus matrix and either HC or SOM for final clustering [1]. Yu et al. used *k*-means as the base clustering on subspace datasets and graph-cut algorithms for the final clustering [2]. Kim used *k*-means as the base algorithm with random multiple number of clusters and applied a graph-cut algorithm for final clustering [3]. The base clustering generates diverse clustering solutions through: 1) generating subspace datasets using gene resampling [1,2,4]; 2) using a single clustering algorithm with random parameter initializations such as selecting a random number of clusters [3,4]; 3) using different clustering algorithms for each base clustering [5]. Some consensus clustering methods used a pairwise similarity matrix of instances to combine multiple clustering solutions [1,2], others used associations between instances and clusters in the consensus matrix [4]. These consensus clustering algorithms usually outperform single clustering algorithms on gene expression datasets [1-4].

Consensus clustering has been used for clustering samples to discover and classify cancer types in cancer microarray data [1-4,6]. It achieved successes in capturing informative patterns from microarray data [1-3]. A well known consensus clustering algorithm, link-based cluster ensemble (LCE) was introduced in [4]. LCE outperforms 10 algorithms tested in [4], specifically, four simple clustering algorithms, three pairwise similarity based consensus clustering algorithms, and three graph-based cluster ensemble techniques. Consensus clustering is also used for clustering genes to identify biologically informative gene clusters [5].

Many studies used prior knowledge in clustering genes [7-13]. These methods are referred as semi-supervised clustering approaches. The results showed that using small amount of prior knowledge was able to significantly improve the clustering results; also the more specific prior knowledge used the better in improving the quality of clustering.

Consensus clustering itself can be considered as unsupervised and improves the robustness and quality of results. Semi-supervised clustering is partially supervised and improves the quality of results in domain knowledge directed fashion. Although there are many consensus clustering and semi-supervised clustering approaches, very few of them used prior knowledge in the consensus clustering. Yu et al. used prior knowledge in assessing the quality of each clustering solution and combining them in a consensus matrix [14]. In this paper, we propose to integrate semi-supervised clustering and consensus clustering, design a new semi-supervised consensus clustering algorithm, and compare it with consensus clustering and semi-supervised clustering algorithms, respectively. In our study, we evaluate the performance of semi-supervised consensus clustering, consensus clustering, semi-supervised clustering and single clustering algorithms using *h*-fold cross-validation. Prior knowledge was used on *h*-1 folds, but not in the testing data. We compared the performance of semi-supervised consensus clustering with other clustering methods.

## Method

Our semi-supervised consensus clustering algorithm (SSCC) includes a base clustering, consensus function, and final clustering. We use semi-supervised spectral clustering (SSC) as the base clustering, hybrid bipartite graph formulation (HBGF) as the consensus function, and spectral clustering (SC) as final clustering in the framework of consensus clustering in SSCC.

### Spectral clustering

The general idea of SC contains two steps: spectral representation and clustering. In spectral representation, each data point is associated with a vertex in a weighted graph. The clustering step is to find partitions in the graph. Given a dataset *X*‒=‒{*x*_*i*_|*i*‒=‒1, …, *n*} and similarity *s*_*i* *j*_‒≥‒0 between data points *x*_*i*_ and *x*_*j*_, the clustering process first construct a similarity graph *G*‒=‒(*V*, *E*), *V*‒=‒{*v*_*i*_}, *E*‒=‒{*e*_*i* *j*_} to represent relationship among the data points; where each node *v*_*i*_ represents a data point *x*_*i*_, and each edge *e*_*i* *j*_ represents the connection between two nodes *v*_*i*_ and *v*_*j*_, if their similarity *s*_*i* *j*_ satisfies a given condition. The edge between nodes is weighted by *s*_*i* *j*_. The clustering process becomes a graph cutting problem such that the edges within the group have high weights and those between different groups have low weights. The weighted similarity graph can be fully connected graph or *t*-nearest neighbor graph. In fully connected graph, the Gaussian similarity function is usually used as the similarity function *s*_*i* *j*_= exp(-∥*x*_*i*_-*x*_*j*_∥^2^/2*σ*^2^), where parameter *σ* controls the width of the neighbourhoods. In *t*-nearest neighbor graph, *x*_*i*_ and *x*_*j*_ are connected with an undirected edge if *x*_*i*_ is among the *t*-nearest neighbors of *x*_*j*_ or vice versa. We used the *t*-nearest neighbours graph for spectral representation for gene expression data.

### Semi-supervised spectral clustering

SSC uses prior knowledge in spectral clustering. It uses pairwise constraints from the domain knowledge. Pairwise constraints between two data points can be represented as *must-links* (in the same class) and *cannot-links* (in different classes). For each pair of *must-link* (*i*,*j*), assign *s*_*i* *j*_ = *s*_*j* *i*_ = 1, For each pair of *cannot-link* (*i*, *j*), assign *s*_*i* *j*_ = *s*_*j* *i*_ = 0.

If we use SSC for clustering samples in gene expression data using *t*-nearest neighbor graph representation, two samples with highly similar expression profiles are connected in the graph. Using *cannot-links* means to change the similarity between the pairs of samples into 0, which breaks edges between a pair of samples in the graph. Therefore, only *must-links* are applied in our study. The details of SSC algorithm is described in Algorithm 1. Given the data points *x*_1_, …, *x*_*n*_, *l* pairwise constraints of *must-link* are generated. The similarity matrix *S* can be obtained using similarity function *s*_*i* *j*_ = exp(-∥*x*_*i*_ - *x*_*j*_∥^2^/2*σ*^2^). *σ* is the scaling parameter for measuring when two points are considered similar, and was calculated according to [15]. Then *S* is modified to be a sparse matrix, only *t* nearest neighbors are kept for each data point in *S*. Then, *l* pairwise constraints are applied in *S*. Steps 5-10 follow normalized spectral clustering algorithm [16,17]. 

### Consensus function

We used LCE ensemble framework in our SSCC adopting HBGF as the consensus function. The cluster ensemble is represented as a graph that consists of vertices and weighted edges. HBGF models both instances and clusters of the ensemble simultaneously as vertices in the graph. This approach retains all information provided by a given ensemble, allowing the similarities among instances and among clusters to be considered collectively in forming the final clustering [18]. More details about LCE can be found in [4].

### Semi-supervised consensus clustering

To make a consensus clustering into a semi-supervised consensus clustering algorithm, prior knowledge can be applied in base clustering, consensus function, or final clustering. Final clustering is usually applied on the consensus matrix generated from base clustering. SSCC uses semi-supervised clustering algorithm SSC for base clustering, does not use prior knowledge either in consensus function or final clustering. Our experiment was performed using *h*-fold cross-validation. The dataset was split into training and testing sets, and the prior knowledge was added to the *h*-1 folds training set. After the final clustering result was obtained, it was evaluated on the testing set alone. The influence of prior knowledge could be assessed in a cross-validation framework.

Our semi-supervised consensus clustering algorithm is described in Algorithm 2. Similar to [4], for a given *n* × *d* dataset of *n* samples and *d* genes, a *n* × *q* data subspace (*q* < *d*) is generated by

(1) $$q=q_{\text{min}}+\lfloor \alpha (q_{\text{max}}-q_{\text{min}})\rfloor$$

*α* ∈ [ 0,1] is a uniform random variable, *q*_*m* *i* *n*_ and *q*_*m* *a* *x*_ are the lower and upper bonds of the subspace. *q*_*m* *i* *n*_ and *q*_*m* *a* *x*_ are set to 0.75*d* and 0.85*d*. Let $\prod =\pi_{1},\ldots ,\pi_{m}$ be a cluster ensemble with *m* clustering solutions. SSC is applied on each subspace dataset to obtain clustering results. We use the fixed number of clusters *k*, each $\pi_{i}=C_{1}^{i},\ldots ,C_{k}^{i}$ is one clustering solution. A basic cluster-association matrix *BM* is generated at first based on the crisp associations between samples and clusters using HBGF, in which there are *n* samples and *m*×*k* clusters. If *x*_*i*_ belongs to a cluster *C*_*j*_, *BM*(*x*_*i*_,*C*_*j*_)=1,*i*=1,…,*n*; *j*=1,…,*g*, otherwise *BM*(*x*_*i*_,*C*_*j*_)=0. Next, a refined cluster-association matrix *RM* is generated from *BM* by estimating new association values in *RM*(*x*_*i*_,*C*_*j*_) if *BM*(*x*_*i*_,*C*_*j*_)=0. *RM*(*x*_*i*_,*C*_*j*_) is the similarity between *C*_*j*_ and other clusters to which *x*_*i*_ probably belongs. The similarity of any clusters in the cluster ensemble is obtained from a weighted graph of clusters. Finally, spectral clustering is applied on *RM* to obtain the final clustering solution. 

## Results

### Selected algorithms

We compared the performance of four algorithms: SSCC, SSC [19], LCE [4], and *k*-means (Table 1). The performance of SSCC was influenced by amount of prior knowledge, consensus function and base clustering. By increasing the amount of prior knowledge, we observed the influence of prior knowledge on SSCC. SSCC uses SSC as the base clustering. By comparing SSCC with SSC on the same amount of prior knowledge, we were able to observe the influence of consensus clustering on SSCC. Same as LCE, SSCC uses HBGF as the consensus function. SSCC became a consensus clustering algorithm when it did not use prior knowledge. *k*-means was used as the baseline algorithm in this study. In both SSCC and LCE, we used subspace and fixed number of clusters, ensemble size of 10, and nearest neighbor size of 5. We implemented SSCC in Matlab and adopted Matlab code of SSC [20], LCE [4] and *k*-means.

**Table 1:** Attributes of four clustering algorithms

**Clustering**	**Type**	**Base**	**Final**	**Consensus**	**Using prior**
**algorithms**		**clustering**	**clustering**	**function**	**knowledge**
*k*-means	Simple clustering	*k*-means	-	-	No
LCE	Consensus clustering	*k*-means	SC	HBGF	No
SSC	Semi-supervised clustering	SC	-	-	Yes
SSCC	Semi-supervised consensus clustering	SSC	SC	HBGF	Yes

### Datasets

All four algorithms were tested with eight cancer gene expression datasets (Table 2). These were processed datasets after removing the non-informative genes and obtained from [21]. Prior knowledge was represented as pairwise constraints generated from class labels. Prior knowledge in the eight datasets was derived from sample class labels. A pair of samples share the same class were given a *must-link* prior knowledge. We used a small amount of prior knowledge to test the effectiveness of SSCC (Table 2).

**Table 2:** Cancer gene expression datasets used in experiments

**Dataset**	**Samples**	**Original**	**Selected**	**Classes**	**Constraints**	**Constraints**
		**probes**	**probes**		**number**	**% in total**
CNS [22]	42	7129	1379	5	20	2.2%
Leukemia1 [23]	72	7129	1877	2	20	0.77%
Leukemia2 [23]	72	7129	1877	3	20	0.77%
Leukemia3 [24]	72	12582	2194	3	20	0.77%
LungCancer [25]	203	12600	1543	5	100	0.48%
St.Jude [26]	248	12625	2526	6	100	0.32%
Multi-Tissue1 [27]	174	12533	1571	10	100	0.66%
Multi-Tissue2 [28]	190	16063	1363	14	100	0.55%

### Performance measures

The performance was measured with normalized mutual information (NMI) [29] and adjusted rand index (ARI) [30]. ARI is often used to assess the performance of clustering samples in gene expression datasets [1-4]. The definition of NMI is described as follows. Let X and Y be the random variables described by the cluster assignments and class labels. *I*(*X*,*Y*) denotes the mutual information between *X* and *Y*; *H*(*X*) and *H*(*Y*) the entropy of *X* and *Y*. NMI is defined by

(2) $$\text{NMI}(X,Y)=\frac{I(X,Y)}{\sqrt{H(X)H(Y)}}$$

### Experimental results

**Figure 1:**
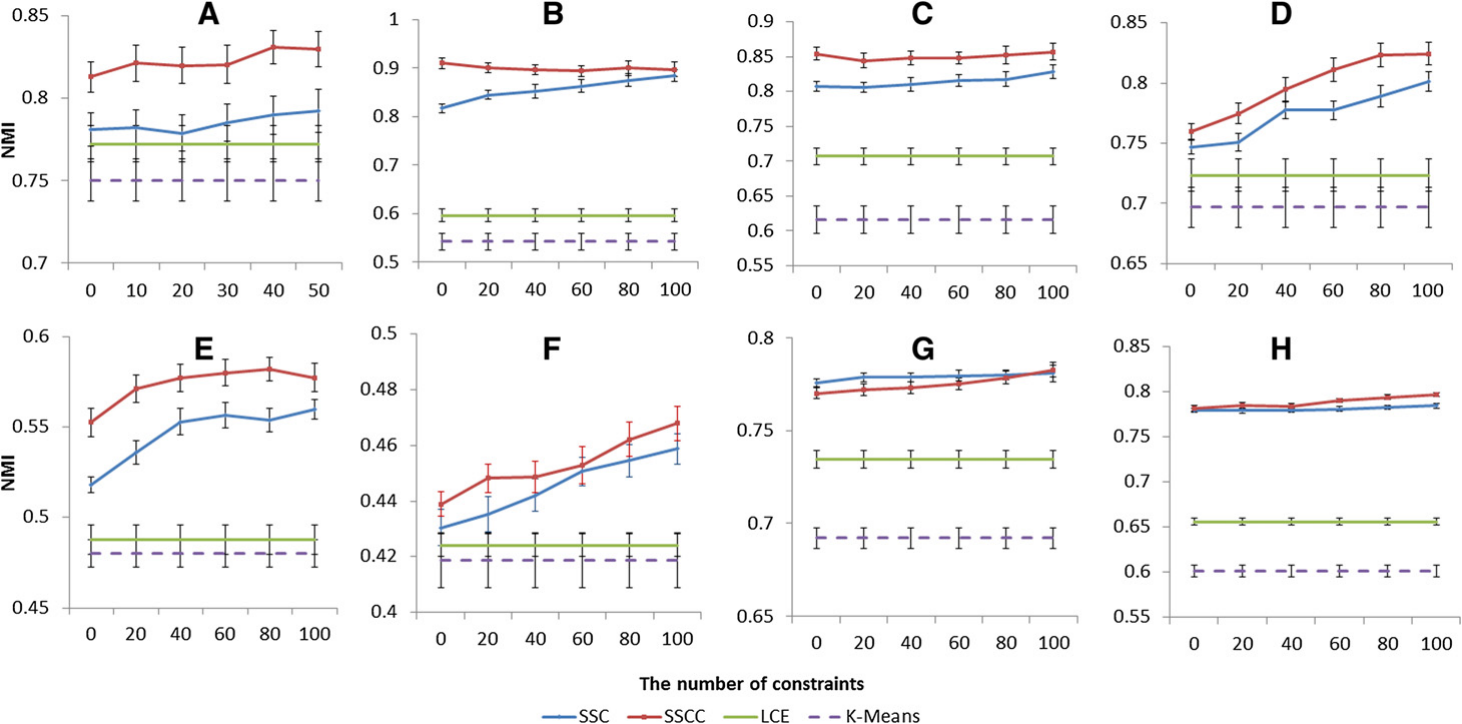
Normalized mutual information with various numbers of constraints on (A) CNS (B) Leukemia1 (C) Leukemia2 (D) Leukemia3 (E) LungCancer (F) St. Jude (G) Multi-Tissue1 (H) Multi-Tissues2 datasets (Error bars show 95% confidence interval).

**Figure 2:**
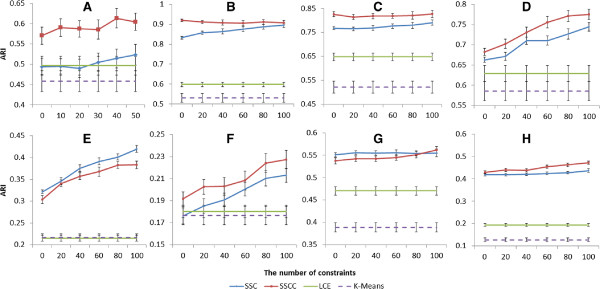
Adjusted rand index with various numbers of constraints on (A) CNS (B) Leukemia1 (C) Leukemia2 (D) Leukemia3 (E) LungCancer (F) St. Jude (G) Multi-Tissue1 (H) Multi-Tissues2 datasets (Error bars show 95% confidence interval).

The experiments were performed by increasing number of pairwise constraints with 5 fold cross validation and 50 runs (Figures 1, 2).

Without prior knowledge, comparisons of SSCC, SSC, LCE and *k*-means was performed by using one-way ANOVA with Bonferroni correction (*p*<0.05) on NMI and ARI (Table 3 and Additional file 1). We used paired t-test (*p*<0.05) to compare SSCC and SSC with prior knowledge on NMI and ARI, respectively. The null hypothesis was that no difference existed between the mean of SSCC and SSC. We used 20 pair-wise constraints for CNS, Leukemia1, Leukemia2 and Leukemia3, but 100 constraints for other 4 datasets (Table 4).

**Table 3:** **Without prior knowledge, comparison among SSCC, SSC, LCE, and *k*-means**

	**NMI**			**ARI**		
	**SSC**	**LCE**	***k*-means**	**SSC**	**LCE**	***k*-means**
SSCC	4/4/0	7/1/0	8/0/0	4/3/1	7/1/0	8/0/0
SSC/SC	-	6/2/0	8/0/0	-	6/2/0	6/2/0
LCE	-	-	6/2/0	-	-	5/3/0

All results are summarized in w/t/l, i.e. the first algorithm wins w times, ties t times and loses l times.

**Table 4:** With prior knowledge, paired t-test for the mean difference between SSCC and SSC

	**NMI**	**ARI**
CNS	0.041*	0.097*
Leukemia1	0.056*	0.053*
Leukemia2	0.094*	0.143*
Leukemia3	0.024*	0.031*
Lungcancer	0.018*	-0.037*
St.Jude	0.009*	0.0144*
MultiTissue1	0.002	0.007
MultiTissue2	0.012*	0.035*
	SSCC vs. SSC	SSCC vs. SSC
w/t/l	7/1/0	6/1/1

*The mean difference (SSCC - SSC) is significant at *p*<0.05 level. The results are summarized in w/t/l, i.e. the first algorithm wins w times, ties t times and loses l times.

Our result clearly demonstrated that consensus clustering and using prior knowledge both contribute to improving the quality of clustering and an integration of both performed even better (Figures 1, 2 and Tables 3, 4). Without injection of prior knowledge, performance of SSCC and SSC were more or less equivalent, but both were significantly better than LCE and *k*-means (Table 3). On the other hand, with injection of prior knowledge, SSCC significantly outperformed SSC (Table 4).

### Parameter analysis

**Figure 3:**
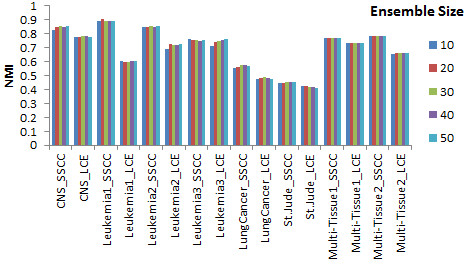
Normalized mutual information of SSCC and LCE with the change of ensemble size on eight datasets.

Ensemble size was one of important parameters that influence SSCC and LCE (Figure 3). SSCC significantly outperformed LCE in all ensemble size settings across the 8 datasets excepting size 40 and 50 on Leukemia3. In some datasets, the performance of SSCC or LCE is improved with the increase of ensemble size from 10 to 20. However, there is no significant improvement in other datasets such as Multi-Tissue1 and Multi-Tissue2. In such case we suggest a small ensemble size, such as 10.

**Figure 4:**
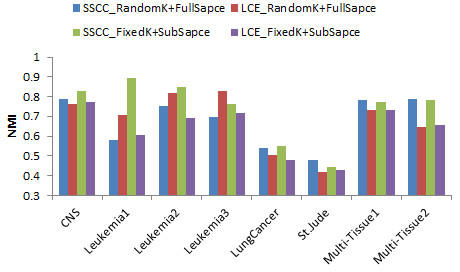
Normalized mutual information of SSCC and LCE with two ensemble types on eight datasets.

Influence of ensemble type appeared to be more obvious (Figure 4). We compared the performance of two ensemble types, “Fixed *k* + Subspace” and “Random *k* + Full-space”, on SSCC and LCE. SSCC outperformed LCE with both ensemble types in majority of the 8 datasets. SSCC with “Fixed *k* + Subspace” appeared to be generally better than other combinations.

**Figure 5:**
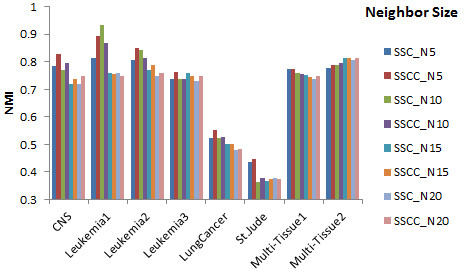
Normalized mutual information of SSC and SSCC with various numbers of neighbor size on eight datasets.

Performance of both SSCC and SSC was significantly influenced by neighborhood size (Figure 5). Without applying prior knowledge, we conducted paired two-tailed t-test (*p*<0.05) between SSCC and SSC under four different *t* values. In majority of the datasets, both algorithms performed better with smaller neighborhood size. Generally, SSCC outperformed SSC.

## Discussion

We compared the performance of SSCC with SSC, LCE and *k*-means and each of our pairwise comparison provides information of the effect of either semi-supervision or consensus clustering. Specifically, comparing LCE with *k*-means reveals the effectiveness of ensemble strategy since *k*-means is used as the base clustering in LCE. Similarly, in comparing SSC with SSCC, we used the same amount of prior knowledge, so actually we compared spectral clustering with consensus clustering. The comparison between SSCC and LCE reveals the effect of semi-supervision under the consensus clustering paradigm.

SSCC significantly outperforms SSC with or without prior knowledge. This clearly shows that consensus clustering algorithms outperform single clustering algorithms in the gene expression datasets. This observation is consistent with [1-4].

We compared SSCC with LCE using the same datasets and same parameter settings. Without considering prior knowledge, the difference between SSCC and LCE is in base clustering, SSCC uses spectral clustering but LCE uses *k*-means. They both use spectral clustering for final clustering (Table 1). Without prior knowledge, SSC becomes SC, and SC outperforms *k*-means in all 8 datasets (Figures 1, 2 and Table 3). This indicates the performance of base clustering has significant influence on results of consensus clustering.

SSCC consists of spectral clustering and LCE. The majority of computational time of spectral clustering spends on finding *t* nearest neighbors [20]. The time complexity of obtaining *t* nearest neighbor sparse matrix is *O*(*n*^2^*d*)+*O*(*n*^2^ log*t*), where *n* is the number of samples, *d* is the number of genes in the graph of spectral clustering. We use the fixed number of cluster *k* in LCE, the time complexity of generating a cluster-association matrix *R* is *O*(*m*^2^*k*^2^+*nmk*)+*O*(*m*^2^*k*^2^*t*^′^+*nmk*), where *m* is ensemble size, and *t*^′^ is the average number of neighbors connecting to one cluster in a network of clusters in final clustering. In SSCC, the complexity of generating *l* pairwise constraints is *O*(*l*). The overall time complexity of SSCC using “Fixed k + subspace” ensemble type is

$$O(l)+O\left(mn^{2}d\right)+O\left(mn^{2}\log t\right)+O\left(m^{2}k^{2}+\text{nmk}\right)+O\left(m^{2}k^{2}t^{\prime }+\text{nmk}\right)$$

Since *n*>*m*, *n*>*k*, *d*>*n*, *d*>*l*, and *d*>*t* in our experiments, the bottle neck of SSCC is to find *t* nearest neighbors with computational time *O*(*mn*^2^*d*). The implementation of spectral clustering is a parallel algorithm [20], so the majority of computational time of SSCC can be reduce to $O\left(\frac{mn^{2}d}{p^{\prime }}\right)$, where *p*^′^ is the number of parallel threads. SSCC is limited to large data set due to the computational complexity of spectral clustering. SSCC can be improved by adopting faster spectral clustering algorithms, which are applicable for data sets with thousands of instances.

Our study provided an insight into the contribution of consensus clustering and semi-supervised clustering to the clustering results. To our knowledge, the Knowledge based Cluster Ensemble (KCE) [14] is the only algorithm using prior knowledge in consensus clustering paradigm for gene expression datasets. Unfortunately, we are unable to directly compare SSCC with KCE because of the unavailability of the software.

Our study uses SSCC for clustering samples. Since the optimal number of clusters (*k* in *k*-means algorithm) and the class label of each sample are known, the prior knowledge is derived from the given class structure. A *must-link* constraint is given to a pair of samples if they are from the same class. For many real applications, we might not know the whole class structure, but most likely we know whether some of samples are in the same class (cluster). We can generate *must-links* between these samples, and prior knowledge is derived from these samples. In these cancer gene expression datasets, we validate the performance of SSCC with the labeled data. The next step would be to apply SSCC for clustering genes for gene function prediction. However, the performance on clustering genes might vary due to two reasons: the quality of prior knowledge and the optimal number of clusters. Pairwise constraints in this study have been generated from class labels of samples in the cancer gene expression datasets and they are true prior knowledge. Prior knowledge in clustering of genes will be known gene functions, and they are partial domain knowledge. A gene may have multiple functions; some functions are inclusive to others as well. For example, a level 6 gene ontology term apoptotic process (GO:0006915) has over ten thousands of gene products and under which at level 7, there are 21 GO terms. Our earlier work shows that more specific (higher level) GO term contribute better to semi-supervised clustering result [13]. Also the description of a certain gene function is based on current knowledge in the domain field. Such domain knowledge is often subject to change. For example, current knowledge of certain existing gene is limited and will gradually be enriched. Therefore, the generated prior knowledge from a pair of genes most likely contains certain noise and subsequently influence the results. The optimal number of clusters is often unknown and a different distance measure would generate a different optimum number of clusters. Therefore, for comparison of semi-supervised clustering algorithms, it is better to use defined prior knowledge, such as the sample labels we used in this paper. When an algorithm considered to be superior over the others, such an algorithm can be used to cluster genes.

In reality, obtaining large amount of prior knowledge for gene expression datasets is difficult. Designing algorithms which work best with a small amount of prior knowledge, such as less than 20 pairwise constraints, will be very useful for clustering microarray data. A study on semi-supervised clustering shows that with small amounts of prior knowledge, search-based approach tends to outperform similarity-based [31]. With larger amounts of labeled data, similarity-based tends to perform better. Combining both approaches outperforms respective individual approaches. SSC is a similarity-based semi-supervised clustering algorithm. The results in Figures 1, 2 show that the performance of SSCC and SSC is slightly improved with small numbers of constraints and significantly improved with increasing numbers of constraints. Our SSCC method presented in this paper is applicable not only to gene expression data, but also to other types of data as long as prior knowledge is provided.

## Conclusions

In this study, we proposed a new semi-supervised consensus clustering method, designed an algorithm, and compared it with another semi-supervised clustering algorithm, a consensus clustering algorithm and a simple clustering algorithm on eight real cancer gene expression datasets. In general, using prior knowledge improves the performance of clustering in gene expression datasets. Consensus clustering is able to reach the goal of maximizing intra-cluster similarity and minimizing inter-cluster similarity. Also, using prior knowledge enhances the high consistency between data partitioning and domain knowledge. A combination of both significantly improves the quality of clustering. SSCC outperforms the semi-supervised clustering algorithm SSC and consensus clustering algorithm LCE in most datasets over various parameter settings, ensemble size and type, with or without prior knowledge. This study demonstrates that SSCC is an effective and robust semi-supervised consensus clustering algorithm with prior knowledge, and also a superior consensus clustering algorithm without prior knowledge.

## Competing interests

The authors declare that they have no competing interests.

## Authors’ contributions

YW conceived and designed the program. YW and YP wrote the paper. Both authors read and approved the final manuscript.

## Supplementary Material

Additional file 1 **Table S1.** Comparision between SSCC, SSC and LCE. Without prior knowledge, part of results of one-way ANOVA with Bonferroni correction for comparison among SSCC, SSC, and LCE. Click here for file

## References

[B1] Monti S Tamayo P Mesirov J Golub T **Consensus clustering: a resampling-based method for class discovery and visualization of gene expression microarray data** Mach Learn 2003 7 91 118 10.1023/A:1023949509487

[B2] Yu Z Wong H Wang H **Graph-based consensus clustering for class discovery from gene expression data** Bioinformatics 2007 7 2888 2896 10.1093/bioinformatics/btm463 17872912

[B3] Kim E Kim S Ashlock D Nam D **Multi-k: accurate classification of microarray subtypes using ensemble k-means clustering** Bioinformatics 2009 7 260 19698124 10.1186/1471-2105-10-260 PMC2743671

[B4] Lam-on N Boongoen T Garett S **LCE: a link-based cluster ensemble method for improved gene expression data analysis** Bioinformatics 2010 7 12 1513 1519 10.1093/bioinformatics/btq226 20444838

[B5] Swift S Tucker A Vinciotti V Martin N Orengo C Liu X Kellam P **Consensus clustering and functional interpretation of gene expression data** Genome Biol 2004 7 R94 10.1186/gb-2004-5-11-r94 15535870 PMC545785

[B6] Simpson TI Armstrong JD Jarman AP **Merged consensus clustering to assess and improve class discovery with microarray data** BMC Bioinformatics 2010 7 590 10.1186/1471-2105-11-590 21129181 PMC3002369

[B7] Pan W **Incorporating gene functions as priors in model-based clustering of microarray gene expression data** Bioinformatics 2006 7 7 795 801 10.1093/bioinformatics/btl011 16434443

[B8] Huang D Pan W **Incorporating biological knowledge into distance based clustering analysis of gene expression data** Bioinformatics 2006 7 10 1259 1268 10.1093/bioinformatics/btl065 16500932

[B9] Costa IG Krause R Opitz L Schliep A **Semi-supervised learning for the identification of syn-expressed genes from fused microarray and in situ image data** BMC Bioinformatics 2007 7 Suppl 10 S3 10.1186/1471-2105-8-S10-S3 18269697 PMC2230504

[B10] Chopra P Kang J Yang J Cho HJ Kim HS Lee MG **Microarray data mining using landmark gene-guided clustering** BMC Bioinformatics 2008 7 92 10.1186/1471-2105-9-92 18267003 PMC2262871

[B11] Dotan-Cohen D Kasif S Melkman AA **Seeing the forest for the trees: using the gene ontology to restructure hierarchical clustering** Bioinformatics 2009 7 14 1789 1795 10.1093/bioinformatics/btp327 19497934 PMC2705235

[B12] Tari L Baral C Kim S **Fuzzy c-means clustering with prior biological knowledge** J Biomed Inf 2009 7 1 74 81 10.1016/j.jbi.2008.05.009 PMC2673503 18595779

[B13] Doan DD Wang Y Pan Y **Utilization of gene ontology in semi-supervised clustering** IEEE Symposium on Computational Intelligence in Bioinformatics and Computational Biology: 2011 2011 Paris, France: IEEE Computer Society Press 1 7

[B14] Yu Z Wong H You J Yang Q Liao H **Knowledge based cluster ensemble for cancer discovery from biomolecular data** IEEE Trans Nanobiosci 2011 7 2 76 85 10.1109/TNB.2011.2144997 21742574

[B15] Zelnik-manor L Perona P **Self-tuning spectral clustering** Advances in Neural Information Processing Systems: 2004 2004 Vancouver, Canada: Cambridge, MA: MIT Press 1601 1608

[B16] Ng AY Jordan MI Weiss Y **On spectral clustering: Analysis and an algorithm** Advances in Neural Information Processing Systems: 2001 2001 Vancouver, Canada: Cambridge, MA: MIT Press 849 856

[B17] Luxburg UV **A tutorial on spectral clustering, statistics and computing** ACM Comput Surv 2007 7 4 395 416

[B18] Fern XZ Brodley CE **Solving cluster ensemble problems by bipartite graph partitioning** Proceedings of the 21st International Conference on Machine Learning: 2003; Banff, Alberta 2003 New York, NY: ACM Press 182 189

[B19] Kamvar SD Klein D Manning CD **Spectral learning** International Joint Conference of Artifficial Intelligence (IJCAI): 2003; Acapulco, Mexico 2003 Palo Alto, CA: AAAI Press 561 566

[B20] Chen W Song Y Bai H Lin C Chang E **Parallel spectral clustering in distributed systems** IEEE Trans Pattern Anal Mach Intell 2011 7 3 568 586 20421667 10.1109/TPAMI.2010.88

[B21] deSouto M Costa I de Araujo D Schliep A Ludermir T **Clustering cancer gene expression data: a comparative study** BMC Bioinformatics 2008 7 497 10.1186/1471-2105-9-497 19038021 PMC2632677

[B22] Pomeroy SL Tamayo P Gaasenbeek M Sturla LM Angelo M McLaughlin ME **Prediction of central nervous system embryonal tumour outcome based on gene expression** Nature 2002 7 436 442 10.1038/415436a 11807556

[B23] Golub T Slonim DK Tamayo P Huard C Gaasenbeek M Mesirov JP **Molecular classification of cancer: class discovery and class prediction by gene expression monitoring** Science 1999 7 5439 531 537 10.1126/science.286.5439.531 10521349

[B24] Armstrong S Staunton J Silverman L Pieters R Boer M Minden M **Mll translocations specify a distinct gene expression profile that distinguishes a unique leukemia** Nat Genet 2002 7 1 41 47 10.1038/ng765 11731795

[B25] Bhattacharjee A Richards WG Staunton J Li C Monti S Vasa P **Classification of human lung carcinomas by mRNA expression profiling reveals distinct adenocarcinoma subclasses** Proc Natl Acad Sci 2001 7 24 13790 13795 10.1073/pnas.191502998 11707567 PMC61120

[B26] Yeoh E Ross ME Shurtleff SA Williams WK Divyen P Rami M Fred GB **Classification, subtype discovery, and prediction of outcome in pediatric acutelymphoblastic leukemia by gene expression profilling** Cancer Cell 2001 7 2 133 143 10.1016/s1535-6108(02)00032-6 12086872

[B27] Su A Welsh J Sapinoso L Kern S Dimitrov P Lapp H **Molecular classification of human carcinomas by use of gene expression signatures** Cancer Res 2001 7 20 7388 7393 11606367

[B28] Ramaswamy S Tamayo P Rifkin R Mukherjee S Yeang C Angelo M **Multiclass cancer diagnosis using tumor gene expression signatures** Proc Natl Acad Sci USA 2001 7 26 15149 15154 10.1073/pnas.211566398 11742071 PMC64998

[B29] Strehl A Ghosh J **Cluster ensembles: a knowledge reuse framework for combining multiple partitions** J Mach Learn Res 2002 7 583 617

[B30] Hubert L Arabie P **Comparing partitions** J Classif 1985 7 1 193 218 10.1007/BF01908075

[B31] Basu S Bilenko M Mooney RJ **Comparing and unifying search-based and similarity-based approaches to semi-supervised clustering** Proceedings of the ICML-2003 Workshop on the Continuum from Labeled to Unlabeled Data in Machine Learning and Data Mining:2003; Washington, DC 2003 Palo Alto, CA: AAAI Press 42 49

