# Supplementary figures and images for: Semi-supervised consensus clustering for gene expression data analysis
Source: BioData Min. 2014 May 8;7:7. doi: 10.1186/1756-0381-7-7 (PMC4036113; doi:10.1186/1756-0381-7-7)

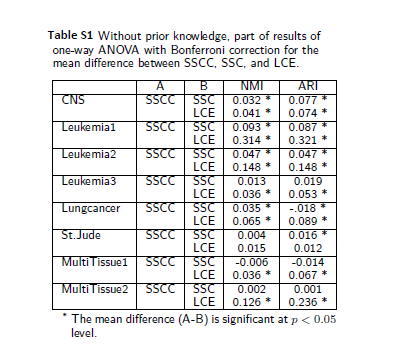

Supplement: Additional file 1 — Table S1. Comparision between SSCC, SSC and LCE. Without prior knowledge, part of results of one-way ANOVA with Bonferroni correction for comparison among SSCC, SSC, and LCE. [file 1756-0381-7-7-S1.png]
